# Proximal regularization of deep residual neural networks applied to high-dimensional genomic data

**DOI:** 10.1093/bib/bbag246

**Published:** 2026-05-25

**Authors:** Yuhua Fan, Ilkka Launonen, Mikko J Sillanpää, Patrik Waldmann

**Affiliations:** Research Unit of Mathematical Sciences, University of Oulu, Pentti Kaiteran katu 1, 90570 Oulu, Finland; Research Unit of Mathematical Sciences, University of Oulu, Pentti Kaiteran katu 1, 90570 Oulu, Finland; Research Unit of Mathematical Sciences, University of Oulu, Pentti Kaiteran katu 1, 90570 Oulu, Finland; Research Unit of Mathematical Sciences, University of Oulu, Pentti Kaiteran katu 1, 90570 Oulu, Finland

**Keywords:** proximal operator, regularization, neural network, high-dimension, genomic data

## Abstract

High-dimensional genomic datasets contain complex patterns shaped by substantial biological noise, which pose major challenges for predictive modeling in genetics and breeding. Residual neural networks (ResNets) provide a powerful framework for capturing nonlinear genomic effects, but often overfit in settings where marker numbers greatly exceed sample sizes. As a solution, a range of regularization methods have been proposed. One promising approach relies on the proximal mapping technique, which is computationally efficient since it can be directly incorporated into the optimization algorithm. However, the performance of ResNets with various convex or non-convex proximal regularizers remains under-explored on high-dimensional data. In this study, we propose an extended stochastic adaptive proximal gradient ResNet method that can handle both convex and non-convex regularizers that range from $L_{0}$ to $L_{\infty }$ and give more analysis of the convergence guarantee for the convex and non-convex regularizers. Moreover, we evaluate the prediction performance in a supervised regression setting on four real high-dimensional genomic datasets from mice, pig, wheat, and loblolly pine. For comparison, we also implement and evaluate traditional sparse linear proximal methods with the same regularizers, as well as LightGBM. Experimental results demonstrate that an 18-layer ResNet with $L_{\frac{1}{2}}$ regularization outperforms other configurations on both mice and pig datasets. For the wheat and loblolly pine data, the 15-layer ResNet $L_{\frac{1}{2}}$ configuration achieves the lowest test mean squared errors and the highest distance correlation (dCor). These findings highlight the effectiveness of the regularized adaptive proximal gradient ResNet method and its potential for prediction tasks on high-dimensional genomic data.

## Introduction

High-dimensional genomic datasets, including single nucleotide polymorphism (SNP) arrays and sequencing-derived markers, contain complex patterns influenced by linkage disequilibrium, epistasis, population structure, and substantial biological noise. These characteristics make predictive modeling challenging, particularly because the number of markers often exceeds the number of samples by several orders of magnitude, and the majority of variants contribute weak or nonlinear effects [1]. ResNets were introduced to address the difficulties associated with training of very deep neural networks [2]. The main innovation of ResNets is skip connections, which enable the network to learn residual functions with reference to the layer inputs instead of learning unreferenced functions. This architecture is beneficial in addressing the vanishing and exploding gradient problems. ResNets have emerged as a powerful tool for nonlinear regression and genomic prediction accuracy across diverse species and traits, demonstrating robustness to extrapolation and handling ”out-of-distribution” data samples effectively [3, 4]. By incorporating shortcut residual connections, it has become possible to train much deeper multilayer perceptrons and convolutional neural networks than was previously possible. ResNets have achieved record low error rates, some important examples include Wide ResNet [5], ResNeXt [6], and PyramidNet [7]. However, the ResNet architecture is on its own not sufficient to reduce the generalization error because of overfitting. This overfitting is particularly pronounced when ResNets are trained on small, noisy, or high-dimensional datasets, where the number of parameters far exceeds the number of available samples.

To improve the performance of ResNets, different regularization methods that place more coherent constraints on the model parameters have been proposed [8–10]. Data augmentation techniques artificially increase the diversity of training data, helping to increase the robustness of the model [11]. Early stopping halts training when the validation loss ceases to improve, preventing the model from overfitting [12]. Label smoothing softens hard labels, preventing overconfidence in predictions and improving generalization [13]. Dropout randomly deactivates neurons during training, forcing networks to learn redundant features, which reduces overfitting [14]. Other widely used regularization methods include stochastic gradient descent [15] and weight decay [16]. Batch normalization techniques allow for smooth gradient flow and stable training [17, 18]. Unfortunately, the regularization effect of these approaches is generally too weak on high-dimensional input data.

High-dimensional data analysis is a rapidly growing field of research that focuses on data with a large number of input variables, often much larger than the sample size. For example, high-throughput measurements in genomics often contain thousands or millions of variables, such as single nucleotide polymorphism (SNP) markers and gene expression data, for each individual observation [19, 20]. When applied to this domain, deep learning models face two big challenges: the inherent noise within the data from biological and environmental factors, and the presence of complex, nonlinear interactions between markers, such as linkage disequilibrium and epistasis, which are difficult for traditional models to capture [21, 22]. To effectively model these complex relationships, a deep architecture is necessary. However, traditional neural networks often suffer from vanishing gradients in deep configurations, making training intractable. ResNets, with their residual connections, offer a solution by enabling the stable training of very deep networks capable of modeling these complex genomic interactions. While deep models are powerful, their high number of parameters can easily lead to severe overfitting on noisy genomic data, a problem that standard regularization methods often fail to address adequately.

In this study, the main contributions can be summarized as follows:

(i) We present an extended stochastic adaptive proximal gradient ResNet framework capable of incorporating a broad range of regularization functions, especially both convex and non-convex proximal regularizers ranging from $L_{0}$ to $L_{\infty }$. This framework is designed to effectively enforce sparsity and control model complexity in high-dimensional settings. We also give details of its convergence to demonstrate its theoretical rationality and guarantee.
(ii) We conduct a systematic and in-depth evaluation of this proposed method on four diverse, high-dimensional genomic datasets (mice, pig, wheat, and loblolly pine). To assess the contribution of the proximal regularization framework, we include non-regularized ResNet as a control baseline, trained with the standard Adam optimizer and no proximal mapping step, using the same architecture, folds, and evaluation procedure as the regularized variants. This comprehensive analysis evaluates the impact of various regularization functions and network depth on predictive accuracy, providing valuable insights into optimal model configurations.
(iii) We compare the performance of regularized ResNet method with both traditional sparse linear proximal methods with the same set of regularisers and the representative gradient boosting algorithm LightGBM [23]. This comparison highlights the superior predictive performance of the proposed deep learning approach on typical high-dimensional genomic datasets.

Unlike previous work that has focused on a limited set of regularizers [24], our framework incorporates a broad range of regularization functions that can enforce specific sparsity constraints, which are particularly beneficial for high-dimensional genomic data [25]. Adaptive proximal gradient methods allow dynamic adjustments to learning rates while simultaneously incorporating regularization terms, effectively balancing model complexity, weight sparsity, and predictive accuracy. This dual capability makes them particularly well suited for high-dimensional datasets, where many input variables are redundant.

## Methods

### Problem formulation

ResNets can be susceptible to overfitting when trained on small, noisy, or high-dimensional datasets due to their deep architecture and large number of parameters [26]. Regularization is an essential strategy to mitigate this issue by imposing constraints on the network’s structure and enhancing generalization [27]. The optimization problem for training networks with regularization can be formalized as 


(1) \begin{align*}& \underset{\theta \in \mathbb{R}^{d}}{\mathrm{minimize}} \quad \mathcal{F}(\theta) = \mathcal{L}(\theta) + \lambda\mathcal{R}(\theta),\end{align*}

where $\theta \in \mathbb{R}^{d}$ denotes the network parameters, $\mathcal{L}(\theta )$ is the empirical loss function, $\lambda> 0$ is a regularization parameter, and $\mathcal{R}(\theta )$ is a data-independent regularizer, which encourages more effective models and leads to a better estimation [28]. Proximal gradient methods are particularly well suited for solving such optimization problems as they incorporate regularization terms, including non-differentiable ones, directly into the training process [29]. These methods can ensure that the network parameters are within reasonable bounds and prevent them from becoming excessively large, which would otherwise have a negative impact on predicting unseen data.

Minimizing the above objective function defined is typically accomplished using stochastic first-order optimization techniques applied to the $k$th random mini-batch of the data: 


(2) \begin{align*}& \underset{\theta \in \mathbb{R}^{d}}{\mathrm{minimize}} \quad \mathcal{F}(\theta) = \sum_{k=0}^{n-1} \mathcal{F}_{k} (\theta).\end{align*}

### Adaptive proximal gradient descent methods for ResNets with various regularizer

Adaptive proximal gradient descent methods [30, 31] adjust the learning rate based on past gradient information, introducing a form of preconditioning by scaling gradients based on their historical magnitudes. By combining proximal updates with adaptive preconditioning, the recently introduced ProxGEN offers a robust and flexible optimization method that addresses complex regularization of both non-smooth and non-convex regularization terms in neural network training [24]. Based on the Adam optimizer [32] and the idea of diagonal pre-conditioners [33], the preconditioner for ProxGEN can be formulated as $ C_{t} = \sqrt{\beta _{2} C_{t-1} + (1-\beta _{2})g_{t}^{2}}$, where $g_{t}$ is the gradient of a stochastic mini-batch at iteration $t$ and $\beta _{2} \in [0,1)$. Given a non-empty proximal mapping 


(3) \begin{align*}& \mathrm{prox}_{f}(\theta) = \arg\min_{z}\left\{f(z) + \dfrac{1}{2}\left\|z - \theta \right\|^{2}\right\},\end{align*}

the update rule for the weight parameters then becomes 


(4) \begin{align*}& \theta_{t+1} = \mathrm{prox}_{\alpha_{t}\lambda\mathcal{R}(\cdot)}^{C_{t} + \delta I}(\theta_{t} - \alpha_{t}(C_{t} + \delta I)^{-1}m_{t}),\end{align*}

where $m_{t} = \beta _{1} m_{t-1} + (1 - \beta _{1}) g_{t}$ represents the first-order momentum, $\theta _{t}$ is the parameter vector at iteration $t$, $\lambda> 0$ is the penalty factor, $\alpha _{t}$ is the adaptive learning rate, and $\theta _{t+1}$ is the new parameter vector at iteration $t+1$. The small constant $\delta $ ensures the positive definiteness of the matrix $C_{t} + \delta I$, which is crucial for the stability of algorithm. The parameter update at each iteration is governed by the gradient of loss function, the regularizer, the learning rate $\alpha _{t}$, and the penalty factor $\lambda $. One of the key challenges with the momentum $m_{t}$ in above equation is the initialization bias that occurs when $m_{t-1}$ is set to zero at the start of optimization. When $g_{t}$ is large, this bias will hinder convergence, especially early in training. The bias correction method in Adam is designed to mitigate this initialization bias using the decay rates $\beta _{1}$ and $\beta _{2}$ for the first and second moments, respectively. Algorithm 1 outlines the full ProxGEN method for a general choice of differentiable loss and closed-form proximal regularization functions.



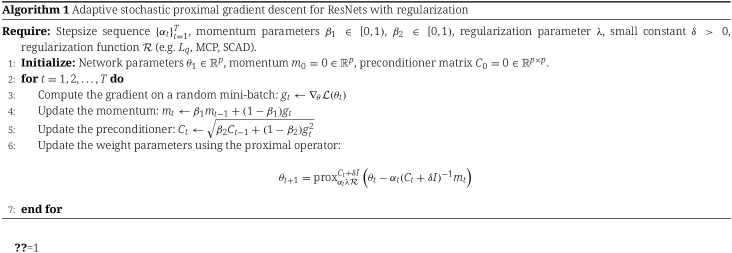



We now take a look at how to generalize the regularization function $\mathcal{R}(\theta )$. The closed-form solution for a general $L_{q}$ regularization problem can be written as 


**Figure 1:**
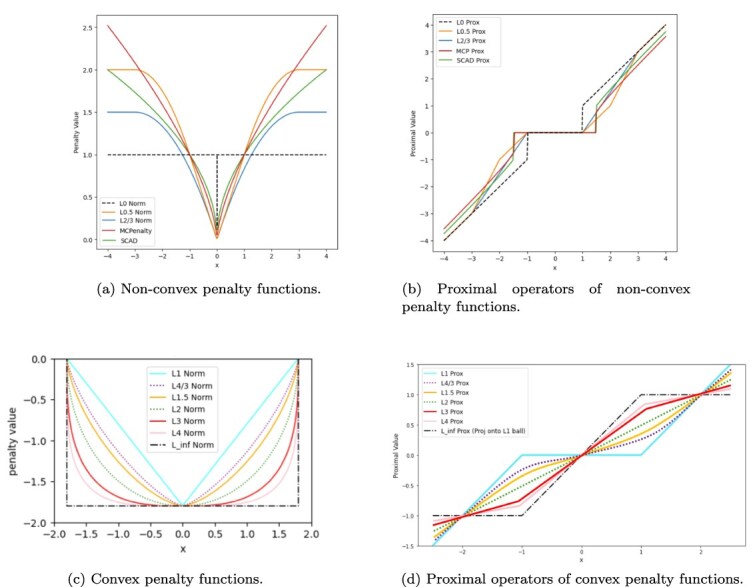
Comparison of non-convex and convex penalty functions and their proximal operators.

(5) \begin{align*}& \hat{\theta} = \arg\min_{\theta}\{(\theta - z)^{2} + \lambda \left\| \theta \right\|^{q}\},\end{align*}

where $\mathcal{R}(\theta ) = \left \| \theta \right \|^{q}$ represents the $L_{q}$ penalty function for $0 \leq q \leq \infty $. We extend the application of sparse $L_{q}$ regularization beyond the conventional range $0 \leq q \leq 1$ to encompass cases where $q> 1$, and therefore end up with regularizers with the following penalty functions: $L_{0}, L_{\frac{1}{2}}, L_{\frac{2}{3}}, L_{1}, L_{\frac{4}{3}}, L_{\frac{3}{2}}, L_{2}, L_{3}, L_{4}$, and $L_{\infty }$. In addition, we also explore the smoothly clipped absolute deviation (SCAD) [34] and the minimax concave penalty (MCP) [35] regularizers. This set of regularizers provides deeper insights into the behavior of various proximal operators and their effects on ResNets in the case of high-dimensional genomic datasets. Figure 1 compares these non-convex and convex penalty functions and their corresponding proximal operators for a single parameter $\theta \in \mathbb{R}$. The mathematical details of the different regularization functions and their proximal operators are provided in the Appendix A. Convergence analysis is given in Appendices B.

### Network architectures

**Figure 2:**
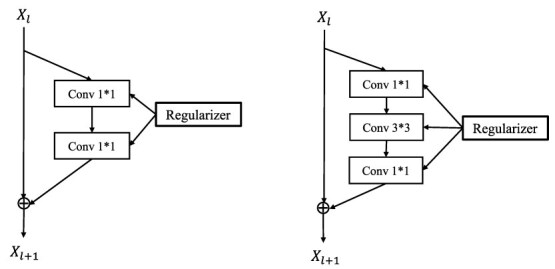
Residual Functions: $\mathcal{E}$ a basic block used in ResNets with 5 to 18 layers (left) and a bottleneck block with three convolutional layers used in ResNets with 20 to 25 layers (right).

We adopt a highly flexible design following ResNets. The network consists of basic block ResNets from 5 layers to 18 layers and of bottleneck block ResNets beyond 18 layers. Figure 2 shows the basic block and the bottleneck block used in this work. A residual block can be formulated as 


(6) \begin{align*}& X_{l+1} = X_{l} + \mathcal{E}(X_{l}, \theta_{l}),\end{align*}

where $X_{l}$ and $X_{l+1}$ are the input and the output of the $l$-th unit in the network, respectively, and $\mathcal{E}$ is a sequence of 1D convolution, batch normalization, and ReLU activation, followed by another 1D convolution and batch normalization. As described in [2], a ResNet consists of sequentially stacked residual blocks. For the basic block, it contains two consecutive $1\times 1$ convolutions with batch normalization and ReLU proceeding convolution, where a stride of 2 is used for dimension reduction in the $1\times 1$ convolution. For the bottleneck block, ResNet utilizes a $1\times 1$ or a $3\times 3$ convolution to adjust the number of channels, where a stride of 1 is applied to the other convolutions to improve computational efficiency. In comparison with previous architectures [5], the use of varying strides and residual blocks mitigates the loss of information at each layer.

## Material

**Mice data:** The first data of this study are the mice data. It is a part of the BGLR package in R [36], but originally comes from the Wellcome Trust (http://gscan.well.ox.ac.uk) and has been used for whole-genome regression in several earlier studies [22, 37]. It consists of genotypes and phenotypes of 1814 mice. Each mouse was genotyped at 10 346 SNPs that were coded as 0, 1, and 2. Here we use two continuous traits, body length and body mass index.

**Pig data:** The largest dataset in this study is the pig data [38], which contain 3534 individuals with high-density genotypes and continuous phenotypes of five anonymized traits. After cleaning some missing data, we finally obtained 2314 samples, where each sample contains 52 843 SNPs. SNPs are encoded as 0, 1, and 2, corresponding to the homozygous reference, heterozygous, and homozygous alternative genotypes, respectively. A small proportion of SNP entries ($\sim $0.13%) contain imputed probabilistic dosage values in the range $(0, 2)$, reflecting uncertainty in the genotype calls. The data were anonymized by randomizing the map order and recording the SNP identities.

**Wheat data:** The wheat dataset originates from CIMMYT’s Global Wheat Program and is a publicly available in the BGLR package [36]. It comprises 599 wheat lines from the CIMMYT Global Wheat Program evaluated in four international environments representing four basic agro-climatic regions (mega-environments). The phenotypic trait used is the 2-year average grain yield of each line in each of the four environments (Env-1, Env-2, Env-3, Env-4), standardized to unit variance within each environment, yielding four prediction targets. The wheat lines were genotyped using 1447 Diversity Array Technology (DArT) markers: 1 indicates the presence of the marker allele and 0 indicates its absence. As a quality control, all the markers with a minor allele frequency below 0.05 were eliminated, and any missing genotypes were imputed once using samples from the marginal distribution of marker genotypes. Following these procedures, the dataset was reduced to 1279 DArT markers.

**Loblolly pine data:** This data is derived from 32 parents representing a wide range of accessions from the Atlantic Coastal Plain, Florida, and the lower Gulf of the USA. Parents were crossed in a circular mating design with additional off-diagonal crosses, resulting in 70 full-sib families with an average of 13.5 individuals per family [39, 40]. It was originally composed of 951 individuals from 61 families that was genotyped using an Illumina Infinium assay [41]. A subset of 4853 SNPs (encoded as 0, 1, 2) were polymorphic and used in our study. There are totally 17 traits in the original data. We selected seven traits: rootnum (Trait 1), rootnumbin (Trait 2), c5c6 (Trait 3), density (Trait 4), lateWood%4 (Trait 5), lignin (Trait 6), and stiffnessTree (Trait 7) due to their high data quality. After cleaning the missing values, we finally got 806 samples and each sample contains 4853 SNPs. The dataset was divided into three subsets—training, validation, and testing with the same percentage as other data used.

## Implementation details

### Baseline models

**Non-regularized ResNet:** To quantify the specific contribution of the proximal regularization framework, we use non-regularized ResNets as a control baselines separately on each of the four datasets. The architecture is identical to that of the regularized variants and each consists of one basic block module with two 1D convolutional layers, batch normalization, ReLU activations, and a shortcut connection, followed by adaptive average pooling and a fully connected output layer. The standard Adam optimizer [32] is used in place of the custom proximal gradient optimizer, with no proximal mapping step applied after each gradient update.

**PGDLM:** We also evaluate a traditional linear model implemented within proximal gradient descent (PGDLM) framework. For PGDLM, we use exactly the same penalty functions as for the regularized ResNets.

**LightGBM:** In earlier evaluations among gradient boosting methods (including XGBoost and CatBoost) and Random Forest, across a wide range of tabular and genomic datasets, it has been found that LighGBM performs very competitive [42–44]. Therefore, we have chosen it as the most competitive nonlinear non-deep-learning baseline for this study. To prevent overfitting, we used $L_{1}$ regularization with the regularization factor tuned over the range $[10^{-6}, 10^{-1}]$ via Bayesian optimization (BO). Performance metrics and evaluation frameworks were aligned with our earlier work [25], enabling direct comparison with previously reported results on the same datasets.

### Training

**Data settings:** The datasets are initially split into a training set (80%) and a held-out test set (20%). Within each of 10 independent repetitions (using unique random seeds), the training set is further partitioned into five folds for cross-validation (CV), defined by splitting individuals (rows) rather than polymorphisms (columns). These CV folds are used exclusively for hyperparameter tuning within a BO loop. Once the optimal hyperparameters are identified, the final model is retrained on the entire 80% training set and evaluated on the held-out test set. To ensure a rigorous and fair comparison, the same CV folds and test partitions are applied across all methods, regularizers, and traits within each repetition.

**Regularized ResNet:** We train regularized ResNets on the four datasets using various regularizers defined by the objective function with convex and non-convex $L_{q}$ regularization: $\mathcal{F}(\theta ) = \mathcal{L}(\theta ) + \lambda \sum _{j=1}^{p} \left | \theta _{j} \right |{}^{q}$, where $0\leq q\leq \infty $. The parameters are trained with the closed-form proximal mappings presented in Appendix A. We explore a range of ResNet architectures aimed for 1D genomic data, including ResNet-5, ResNet-10, ResNet-15, ResNet-18, ResNet-20, ResNet-25. Each model consists of basic block modules with convolutional layers, batch normalization, and a final fully connected linear output layer. To incorporate regularization, we implement a custom optimizer, which extends the standard PyTorch class. This optimizer combines an adaptive gradient step, similar to the Adam optimizer, with a subsequent proximal mapping step. Our implementation uses a momentum-like update with $\beta _{1} = 0.1$ and $\beta _{2} = 0.999$. After the gradient update, the optimizer applies a proximal operator to each weight, defined by the specific penalty functions. During training, the validation mean squared error (MSE) is monitored closely. If models fail to adequately capture the data complexity, increasing the depth of the network is considered. Conversely, if overfitting persists despite regularization efforts, a shallower network with fewer layers is evaluated. The same 10-repeat evaluation procedure described above is applied to all regularized ResNet variants, ensuring that all comparisons between the baseline and regularized models are based on identical data partitions. All features are normalized to zero mean and unit variance prior to training.

### Hyperparameter optimization

**Figure 3:**
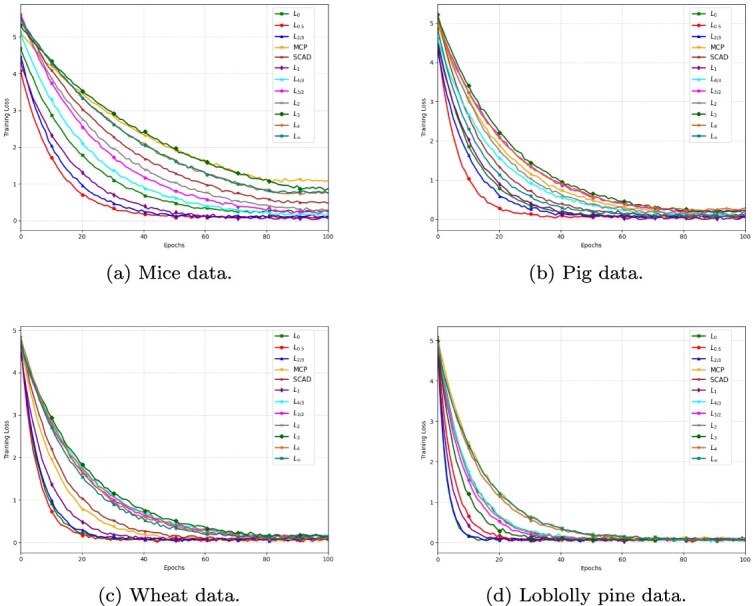
Training loss curves demonstrating various regularization effects on four datasets: ResNet-18 for the mice and pig data and ResNet-15 for the wheat and loblolly pine data.

**Figure 4:**
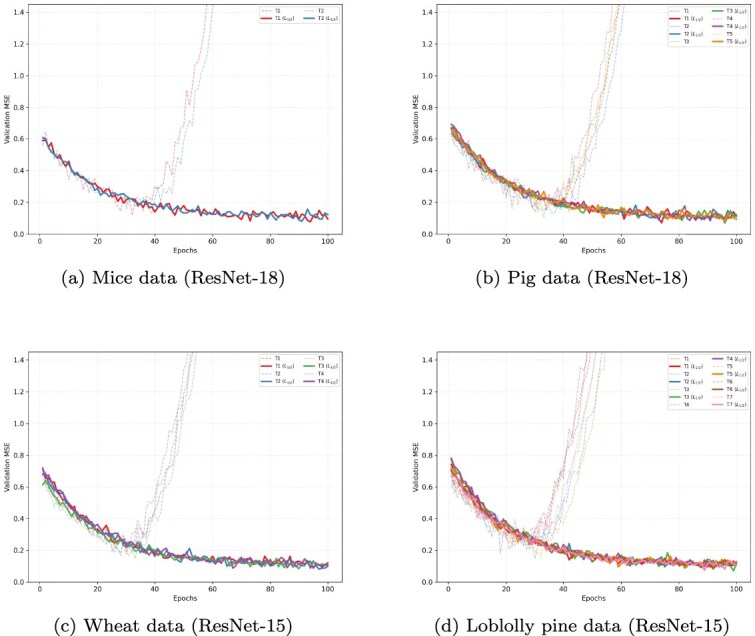
Validation MSE trajectories across four genomic datasets: the solid lines regularized ResNet with $L_\frac{1}{2}$ regularizer, while the dashed lines show unregularized ResNet.

BO is employed to identify the optimal configurations for both regularized and non-regularized ResNets, with performance measured by the average validation MSE across five folds. The BO search space for each regularized ResNet encompasses three hyperparameters: (i) learning rate: continuous range $[10^{-4}, 10^{-2}]$; (ii) regularization parameter $\lambda $: continuous range $[10^{-3}, 10^{2}]$; and (iii) batch size: discrete set $\{32, 64, 128\}$. We utilized the Tree-structured Parzen Estimator (TPE) algorithm [45], implemented via the Hyperopt library [46], to ensure efficient exploration of the parameter space. For the non-regularized (vanilla) ResNet, the search space is restricted to the learning rate and batch size, following the same TPE-based BO procedure. The Adam optimizer is used with default moment parameters ($\beta _{1} = 0.9$, $\beta _{2} = 0.999$, and $\epsilon = 10^{-8}$). All models are subsequently retrained for 50 epochs using the best identified hyperparameters and evaluated on the held-out test set. Figure 3 illustrates the training loss trajectories, while Fig. 4 provides a comparison of validation MSE between the non-regularized and $L_\frac{1}{2}$-regularized models across all datasets (using ResNet-18 for Mice/Pig and ResNet-15 for Wheat/Loblolly Pine).

### Evaluation metrics

The reported test MSE and distance correlation (dCor) values represent the mean and standard deviation calculated across 10 independent repetitions. We also calculate the Wilcoxon signed-rank test [47, 48] to evaluate the statistical significance of performance differences between the regularized variants and the non-regularized baseline. This test is applied to the 10 paired test MSE values per trait per data. For the sparsity-inducing regularizers, the optimal solution of the weights $\mathbf{w}^{*}$ will be sparse. The sparsity is calculated as 


(1) \begin{align*}& \mathrm{sparsity} = \frac{\text{The number of non-zeros entries of } \mathbf{w}^{*}}{\text{Dimension of } \mathbf{w}^{*}} = \frac{||\mathbf{w^{*}}||_{0}}{d}.\end{align*}

### Parallel computing

All models are implemented using the PyTorch framework, and experiments are run on five NVIDIA GPUs to accelerate training. Parallelization across multiple folds is employed during CV to enhance computational efficiency. The final model configurations are determined by running BO for a maximum of 100 iterations, with convergence considered achieved when the improvement in validation MSE was less than $10^{-5}$ for five consecutive iterations.

## Results and discussion

### Comparison of adaptive proximal methods on different datasets

**Mice data:** From Table 1, it is evident that the choice of regularizer significantly impacts performance. The $L_{\frac{1}{2}}$ regularizer consistently outperformed all other methods, achieving the lowest test MSE and highest distance correlation (dCor) values. For Trait 1, it yielded an MSE of $0.133 \pm 0.002$ and a dCor of $0.733 \pm 0.001$. Similarly, for Trait 2, it produced the lowest MSE of $0.134 \pm 0.005$ and the highest dCor of $0.355 \pm 0.004$. This demonstrates that the $L_{\frac{1}{2}}$ penalty is highly effective for this genomic dataset. The performance of other regularizers varied, but the non-convex regularizers like MCP and SCAD, and those with higher $L_{q}$ norms (e.g. $L_{\frac{3}{2}}$, $L_{3}$, $L_{\infty }$), performed worse than $L_{\frac{1}{2}}$ and $L_{2}$. For instance, MCP and SCAD yielded higher MSE and lower dCor values for both traits compared with $L_{\frac{1}{2}}$. The ResNet-18 models with any of the tested regularizers significantly outperformed the baseline methods, non-regularized ResNet, PGDLM and LightGBM. The PGDLM with $L_{\frac{1}{2}}$ baseline had a test MSE of $0.177\, \pm\, 0.005$ for Trait 1, which is notably higher than the $0.133\, \pm\, 0.002$ achieved by the ResNet-18 with the same regularizer. Similarly, LightGBM with $L_{1}$ performed the worst among all tested models, with a test MSE of $0.194 \,\pm\, 0.006$ for Trait 1 and $0.203\, \pm\, 0.007$ for Trait 2. For the non-regularized ResNet, we can clearly see that the results are worse than the weakest regularized variant, confirming that the performance gains of the regularized models are attributable to the proximal regularization framework rather than to the ResNet architecture itself. The difference between the ResNet architectures and PGDLM and LightGBM highlights the substantial advantage of deep learning-based ResNet approach over both traditional linear models and tree-based methods for this specific genomic prediction task. The experimental results for the remaining ResNet architectures (ranging from ResNet-5 to ResNet-25) on the mice dataset are provided in Supplementary Material S1.

**Table 1:** Performance comparison among ResNet-18 with different regularizers and other baselines on the mice data. Test MSE (mean with stddev) and dCor (mean with stddev). The **bold** values indicate the regularizer yielding the lowest test MSE and the highest distance correlation values. Only the best-performing regularizer for PGDLM is presented

**Method**	**Regularizer**	**Trait 1**		**Trait 2**	
		**MSE**	**dCor**	**MSE**	**dCor**
ResNet-18	$L_{0}$	0.138 (0.005)	0.573 (0.003)	0.141 (0.003)	0.272 (0.002)
	$L_{\frac{1}{2}}$	**0.133 (0.002)**	**0.733 (0.001)**	**0.134 (0.005)**	**0.355 (0.004)**
	$L_{\frac{2}{3}}$	0.134 (0.007)	0.701 (0.011)	0.136 (0.005)	0.301 (0.001)
	MCP	0.142 (0.013)	0.551 (0.001)	0.146 (0.003)	0.264 (0.002)
	SCAD	0.144 (0.003)	0.542 (0.007)	0.147 (0.006)	0.261 (0.005)
	$L_{1}$	0.136 (0.005)	0.637 (0.003)	0.139 (0.008)	0.280 (0.004)
	$L_{\frac{4}{3}}$	0.136 (0.014)	0.592 (0.002)	0.139 (0.013)	0.277 (0.002)
	$L_{\frac{3}{2}}$	0.136 (0.007)	0.554 (0.003)	0.140 (0.006)	0.265 (0.001)
	$L_{2}$	0.137 (0.017)	0.642 (0.001)	0.141 (0.007)	0.282 (0.003)
	$L_{3}$	0.139 (0.005)	0.650 (0.005)	0.143 (0.013)	0.296 (0.001)
	$L_{4}$	0.139 (0.010)	0.673 (0.004)	0.142 (0.007)	0.297 (0.003)
	$L_{\infty }$	0.138 (0.003)	0.605 (0.001)	0.141 (0.005)	0.277 (0.005)
ResNet-18	-	0.152 (0.007)	0.566 (0.003)	0.157 (0.004)	0.266 (0.007)
PGDLM	$L_{\frac{1}{2}}$	0.177 (0.005)	0.564 (0.005)	0.185 (0.014)	0.269 (0.002)
LightGBM	$L_{1}$	0.194 (0.006)	0.550 (0.003)	0.203 (0.007)	0.262 (0.002)

**Pig data:** The pig dataset is the largest among the datasets used in this study, comprising five traits. As shown in Table 2, we observed a significant performance improvement in test MSE and distance correlation (dCor) when increasing the ResNet depth from 5 to 18 layers across all five traits. For instance, using the $L_{\frac{1}{2}}$ regularizer, the test MSE for Trait 1 decreased from $0.140 \pm 0.003$ (ResNet-5) to $0.129 \pm 0.005$ (ResNet-18). This trend highlights that deeper networks are better at learning complex feature representations in larger genomic datasets, leading to superior prediction performance. However, increasing the network depth beyond 18 layers offered only marginal gains. For example, for Trait 4 with the $L_{\frac{1}{2}}$ regularizer, the MSE showed a small improvement from $0.131 \pm 0.004$ (ResNet-18) to $0.130 \pm 0.001$ (ResNet-25), demonstrating a point of diminishing returns.

**Table 2:** Performance comparison among ResNet-18 with different regularizers and other baselines on the pig data. Test MSE (mean with stddev) and dCor (mean with stddev). **Bold** values indicate the regularizer yielding the lowest test MSE and the highest distance correlation values. Only the best-performing regularizer for PGDLM is presented

**Method**	**Regularizer**	**Trait 1**		**Trait 2**		**Trait 3**		**Trait 4**		**Trait 5**	
		**MSE**	**dCor**	**MSE**	**dCor**	**MSE**	**dCor**	**MSE**	**dCor**	**MSE**	**dCor**
ResNet-18	$L_{0}$	0.136 (0.003)	0.801 (0.002)	0.137 (0.001)	0.753 (0.004)	0.136 (0.002)	0.793 (0.003)	0.132 (0.004)	0.772 (0.002)	0.144 (0.003)	0.825 (0.005)
	$L_{\frac{1}{2}}$	**0.129 (0.005)**	**0.901 (0.003)**	**0.130 (0.004)**	**0.924 (0.001)**	**0.129 (0.004)**	**0.910 (0.005)**	**0.126 (0.001)**	**0.982 (0.004)**	**0.133 (0.002)**	**0.941 (0.001)**
	$L_{\frac{2}{3}}$	0.131 (0.002)	0.893 (0.002)	0.132 (0.005)	0.904 (0.004)	0.131 (0.003)	0.877 (0.002)	0.127 (0.003)	0.950 (0.001)	0.135 (0.001)	0.902 (0.004)
	MCP	0.137 (0.004)	0.771 (0.003)	0.138 (0.003)	0.721 (0.003)	0.137 (0.004)	0.765 (0.002)	0.134 (0.001)	0.752 (0.004)	0.144 (0.003)	0.791 (0.004)
	SCAD	0.138 (0.002)	0.766 (0.005)	0.139 (0.004)	0.713 (0.003)	0.138 (0.002)	0.767 (0.006)	0.134 (0.005)	0.749 (0.003)	0.145 (0.003)	0.782 (0.012)
	$L_{1}$	0.135 (0.001)	0.843 (0.002)	0.136 (0.002)	0.821 (0.007)	0.135 (0.003)	0.827 (0.008)	0.132 (0.002)	0.816 (0.002)	0.143 (0.001)	0.852 (0.009)
	$L_{\frac{4}{3}}$	0.135 (0.002)	0.802 (0.003)	0.136 (0.004)	0.787 (0.002)	0.135 (0.005)	0.806 (0.002)	0.132 (0.002)	0.784 (0.003)	0.143 (0.001)	0.830 (0.001)
	$L_{\frac{3}{2}}$	0.136 (0.002)	0.793 (0.002)	0.137 (0.002)	0.743 (0.004)	0.136 (0.002)	0.772 (0.004)	0.132 (0.003)	0.763 (0.004)	0.144 (0.002)	0.813 (0.002)
	$L_{2}$	0.133 (0.003)	0.863 (0.003)	0.134 (0.003)	0.854 (0.003)	0.133 (0.004)	0.847 (0.003)	0.130 (0.001)	0.852 (0.003)	0.140 (0.005)	0.861 (0.003)
	$L_{3}$	0.132 (0.003)	0.841 (0.002)	0.133 (0.002)	0.873 (0.004)	0.132 (0.003)	0.853 (0.003)	0.128 (0.001)	0.872 (0.006)	0.136 (0.003)	0.870 (0.002)
	$L_{4}$	0.132 (0.002)	0.886 (0.003)	0.132 (0.003)	0.873 (0.005)	0.132 (0.004)	0.871 (0.004)	0.127 (0.003)	0.920 (0.002)	0.136 (0.001)	0.881 (0.002)
	$L_{\infty }$	0.134 (0.002)	0.854 (0.002)	0.135 (0.001)	0.779 (0.004)	0.134 (0.002)	0.813 (0.002)	0.131 (0.003)	0.784 (0.002)	0.142 (0.001)	0.791 (0.004)
ResNet-18	-	0.162 (0.003)	0.791 (0.002)	0.167 (0.004)	0.701 (0.014)	0.165 (0.001)	0.751 (0.006)	0.159 (0.005)	0.783 (0.007)	0.168 (0.003)	0.801 (0.004)
PGDLM	$L_{\frac{1}{2}}$	0.177 (0.005)	0.802 (0.002)	0.185 (0.014)	0.801 (0.004)	0.179 (0.010)	0.811 (0.004)	0.171 (0.002)	0.802 (0.005)	0.188 (0.008)	0.837 (0.003)
LightGBM	$L_{1}$	0.194 (0.006)	0.692 (0.003)	0.203 (0.007)	0.653 (0.007)	0.198 (0.011)	0.711 (0.003)	0.189 (0.005)	0.633 (0.007)	0.211 (0.009)	0.681 (0.005)

From Table 2, it is clear that the $L_{\frac{1}{2}}$ regularizer consistently delivered the best performance on ResNet-18 for all five traits, achieving the lowest test MSE and highest dCor. For instance, for Trait 4, it produced the lowest MSE of $0.126 \pm 0.001$ and the highest dCor of $0.982 \pm 0.004$. This shows that the pig data, with their larger size, benefit more from the sparsity properties induced by the $L_{\frac{1}{2}}$ penalty. While performance differences among regularizers narrowed in deeper networks, the $L_{\frac{1}{2}}$ penalty remained the most effective. We can clearly see that all ResNet-18 models with regularization outperformed the baseline methods. The PGDLM with $L_{\frac{1}{2}}$ model and LightGBM showed significantly higher MSE and lower dCor values. The non-regularized ResNet-18 also consistently outperformed LightGBM across all five traits on pig data. This confirms the efficiency of adaptive proximal gradient descent for ResNet. The experimental results for the various ResNet architectures (ResNet-5 through ResNet-25) evaluated on the pig dataset are provided in Supplementary Material S2.

**Wheat data:** The wheat dataset, which consists of four distinct traits, was analyzed using various regularizers applied to ResNet architectures of different depths. As detailed in Table 3, among the ResNet architectures we tested (ResNet-5, ResNet-10, ResNet-15, ResNet-18, ResNet-20, and ResNet-25), ResNet-15 yielded the best overall performance, demonstrating an optimal balance between model complexity and predictive power for this dataset. Deeper models like ResNet-18 and ResNet-25 did not offer significant performance improvements.

**Table 3:** Performance comparison among ResNet-15 with different regularizers and other baselines on the wheat data. Test MSE (mean with stddev) and dCor (mean with stddev). The **bold** values indicate the regularizer yielding the lowest test MSE and the highest distance correlation values. Only the best-performing regularizer for PGDLM is presented

**Method**	**Regularizer**	**Trait 1**		**Trait 2**		**Trait 3**		**Trait 4**	
		**MSE**	**dCor**	**MSE**	**dCor**	**MSE**	**dCor**	**MSE**	**dCor**
ResNet-15	$L_{0}$	0.142 (0.001)	0.719 (0.004)	0.137 (0.003)	0.761 (0.001)	0.134 (0.005)	0.619 (0.002)	0.135 (0.002)	0.742 (0.002)
	$L_{\frac{1}{2}}$	**0.132 (0.002)**	**0.854 (0.003)**	**0.131 (0.002)**	**0.853 (0.001)**	**0.130 (0.003)**	**0.737 (0.002)**	**0.130 (0.002)**	**0.819 (0.004)**
	$L_{\frac{2}{3}}$	0.135 (0.001)	0.816 (0.003)	0.132 (0.005)	0.850 (0.001)	0.132 (0.003)	0.722 (0.002)	0.132 (0.002)	0.792 (0.002)
	MCP	0.143 (0.005)	0.765 (0.004)	0.137 (0.003)	0.742 (0.004)	0.135 (0.002)	0.592 (0.005)	0.136 (0.002)	0.721 (0.004)
	SCAD	0.144 (0.005)	0.761 (0.002)	0.138 (0.004)	0.728 (0.005)	0.136 (0.001)	0.591 (0.002)	0.137 (0.002)	0.720 (0.002)
	$L_{1}$	0.140 (0.003)	0.743 (0.006)	0.136 (0.001)	0.807 (0.004)	0.134 (0.001)	0.621 (0.002)	0.134 (0.003)	0.771 (0.003)
	$L_{\frac{4}{3}}$	0.142 (0.002)	0.724 (0.002)	0.136 (0.003)	0.772 (0.002)	0.134 (0.004)	0.621 (0.003)	0.134 (0.001)	0.773 (0.002)
	$L_{\frac{3}{2}}$	0.142 (0.001)	0.716 (0.004)	0.137 (0.001)	0.756 (0.003)	0.134 (0.002)	0.608 (0.002)	0.135 (0.003)	0.753 (0.005)
	$L_{2}$	0.139 (0.004)	0.757 (0.002)	0.135 (0.003)	0.822 (0.002)	0.133 (0.002)	0.653 (0.001)	0.134 (0.001)	0.776 (0.003)
	$L_{3}$	0.136 (0.002)	0.763 (0.003)	0.133 (0.003)	0.836 (0.005)	0.133 (0.002)	0.704 (0.002)	0.134 (0.001)	0.774 (0.003)
	$L_{4}$	0.135 (0.005)	0.775 (0.002)	0.132 (0.001)	0.841 (0.002)	0.133 (0.002)	0.703 (0.002)	0.133 (0.002)	0.784 (0.001)
	$L_{\infty }$	0.140 (0.002)	0.732 (0.002)	0.135 (0.001)	0.791 (0.002)	0.133 (0.003)	0.638 (0.001)	0.134 (0.003)	0.765 (0.003)
ResNet-15	-	0.163 (0.004)	0.631 (0.006)	0.160 (0.004)	0.673 (0.001)	0.151 (0.006)	0.611 (0.006)	0.154 (0.002)	0.663 (0.005)
PGDLM	$L_{\frac{1}{2}}$	0.177 (0.005)	0.501 (0.007)	0.185 (0.014)	0.483 (0.006)	0.179 (0.010)	0.571 (0.006)	0.171 (0.012)	0.560 (0.001)
LightGBM	$L_{1}$	0.194 (0.006)	0.475 (0.003)	0.203 (0.007)	0.461 (0.002)	0.198 (0.011)	0.503 (0.004)	0.189 (0.005)	0.511 (0.007)

The results show that the $L_{\frac{1}{2}}$ regularizer consistently outperformed all other regularization methods, achieving the lowest test MSE and highest distance correlation (dCor) values across all four traits. For Trait 1, it yielded a test MSE of $0.132 \pm 0.002$; for Trait 2, $0.131 \pm 0.002$; for Trait 3, $0.130 \pm 0.003$; and for Trait 4, $0.130 \pm 0.002$. This consistent superiority suggests that the $L_{\frac{1}{2}}$ penalty provides effective model generalization and is particularly well suited for the complex, high-dimensional data in this genomic prediction task. While the performance differences between the regularizers were not large, several observations can be made. Regularizers such as SCAD and MCP, and those with higher $L_{q}$ norms like $L_{\frac{4}{3}}$ and $L_{\frac{3}{2}}$, consistently resulted in higher test MSEs compared with $L_{\frac{1}{2}}$ and $L_{2}$. For instance, SCAD and MCP yielded test MSEs of $0.144 \pm 0.005$ and $0.143 \pm 0.005$, respectively, for Trait 1, which are notably higher than the $0.132 \pm 0.002$ achieved by $L_{\frac{1}{2}}$. This shows the importance of selecting regularizer to the specific characteristics of data. Furthermore, the performance of $L_{0}$, $L_{\frac{2}{3}}$, and $L_{\frac{4}{3}}$ was similar across traits and slightly inferior to $L_{\frac{1}{2}}$. The non-regularized ResNet-15 achieved higher test MSE and lower test dCor than all regularized variants. Both PGDLM and LightGBM performed substantially worse than all ResNet variants, regularized or not, with PGDLM achieving an MSE of $0.177 \pm 0.005$ and a lower dCor of $0.501 \pm 0.007$ for Trait 1, and LightGBM performing worst overall with MSE values ranging from $0.194 \pm 0.006$ to $0.203 \pm 0.007$ across traits, further confirming the advantage of deep learning-based approaches for this dataset. Full experimental results for the various ResNet architectures (ResNet-5, 10, 18, 20, and 25) evaluated on the wheat dataset are provided in Supplementary Material S3.

**Loblolly pine data:** The analysis of loblolly pine dataset, which includes seven traits, revealed that ResNet-15 provided the best overall performance among the architectures we tested (ResNet-10, ResNet-15, ResNet-18, ResNet-20, and ResNet-25). This finding is consistent with our results for the wheat data, and suggests that an intermediate network depth strikes the right balance between model capacity and generalization for this genomic prediction tasks. As shown in Table 4, the $L_{\frac{1}{2}}$ regularizer consistently achieved the lowest test MSE and highest dCor across all traits. The test MSE for $L_{\frac{1}{2}}$ ranged from $0.139 \pm 0.005$ (Trait 4) to $0.145 \pm 0.006$ (Trait 7), outperforming all other regularizers. This outcome reinforces the prior observations from the mice and wheat datasets, highlighting the superior effectiveness of the $L_{\frac{1}{2}}$ penalty in this context.

**Table 4:** Performance comparison among ResNet-15 with different regularizers and other baselines on the loblolly pine data. Test MSE (mean with stddev) and dCor (mean with stddev). **Bold** values indicate the regularizer yielding the lowest test MSE and the highest distance correlation values. Only the best-performing regularizer for PGDLM is presented

**Model**	**Reg.**	**Trait 1**		**Trait 2**		**Trait 3**		**Trait 4**		**Trait 5**		**Trait 6**		**Trait 7**	
		**MSE**	**dCor**	**MSE**	**dCor**	**MSE**	**dCor**	**MSE**	**dCor**	**MSE**	**dCor**	**MSE**	**dCor**	**MSE**	**dCor**
ResNet-15	$L_{0}$	0.146 (0.003)	0.735 (0.003)	0.146 (0.007)	0.461 (0.002)	0.145 (0.006)	0.508 (0.003)	0.143 (0.007)	0.531 (0.003)	0.145 (0.009)	0.551 (0.003)	0.148 (0.005)	0.681 (0.003)	0.148 (0.004)	0.702 (0.003)
	$L_{\frac{1}{2}}$	**0.141 (0.007)**	**0.883 (0.002)**	**0.145 (0.003)**	**0.521 (0.003)**	**0.140 (0.005)**	**0.656 (0.001)**	**0.139 (0.005)**	**0.637 (0.002)**	**0.140 (0.005)**	**0.617 (0.006)**	**0.143 (0.009)**	**0.753 (0.004)**	**0.145 (0.006)**	**0.791 (0.003)**
	$L_{\frac{2}{3}}$	0.143 (0.004)	0.833 (0.002)	0.145 (0.007)	0.504 (0.002)	0.143 (0.007)	0.617 (0.003)	0.142 (0.007)	0.622 (0.003)	0.143 (0.006)	0.595 (0.008)	0.145 (0.003)	0.743 (0.001)	0.146 (0.002)	0.758 (0.003)
	MCP	0.151 (0.011)	0.691 (0.003)	0.156 (0.017)	0.442 (0.009)	0.150 (0.007)	0.473 (0.003)	0.146 (0.003)	0.515 (0.001)	0.148 (0.007)	0.532 (0.002)	0.155 (0.004)	0.665 (0.003)	0.157 (0.002)	0.681 (0.001)
	SCAD	0.154 (0.007)	0.690 (0.002)	0.158 (0.002)	0.442 (0.004)	0.151 (0.002)	0.470 (0.003)	0.148 (0.003)	0.509 (0.002)	0.149 (0.006)	0.531 (0.004)	0.156 (0.007)	0.659 (0.002)	0.160 (0.002)	0.681 (0.002)
	$L_{1}$	0.146 (0.002)	0.761 (0.002)	0.147 (0.004)	0.471 (0.002)	0.145 (0.006)	0.543 (0.004)	0.144 (0.007)	0.542 (0.002)	0.144 (0.003)	0.573 (0.003)	0.146 (0.002)	0.703 (0.002)	0.148 (0.017)	0.736 (0.001)
	$L_{\frac{4}{3}}$	0.147 (0.016)	0.740 (0.001)	0.149 (0.003)	0.465 (0.001)	0.146 (0.005)	0.511 (0.002)	0.145 (0.002)	0.531 (0.003)	0.146 (0.016)	0.561 (0.001)	0.147 (0.007)	0.686 (0.003)	0.150 (0.002)	0.717 (0.005)
	$L_{\frac{3}{2}}$	0.148 (0.002)	0.712 (0.003)	0.151 (0.001)	0.450 (0.003)	0.148 (0.007)	0.477 (0.005)	0.146 (0.007)	0.523 (0.003)	0.147 (0.002)	0.540 (0.003)	0.149 (0.016)	0.677 (0.001)	0.153 (0.003)	0.692 (0.004)
	$L_{2}$	0.145 (0.011)	0.773 (0.002)	0.147 (0.009)	0.482 (0.010)	0.144 (0.007)	0.566 (0.002)	0.143 (0.006)	0.545 (0.002)	0.144 (0.015)	0.581 (0.001)	0.146 (0.002)	0.712 (0.004)	0.147 (0.004)	0.736 (0.002)
	$L_{3}$	0.151 (0.003)	0.790 (0.007)	0.154 (0.003)	0.491 (0.005)	0.150 (0.015)	0.581 (0.002)	0.146 (0.007)	0.559 (0.002)	0.148 (0.003)	0.591 (0.003)	0.153 (0.017)	0.727 (0.007)	0.155 (0.003)	0.742 (0.003)
	$L_{4}$	0.144 (0.001)	0.812 (0.012)	0.146 (0.007)	0.502 (0.002)	0.143 (0.008)	0.607 (0.001)	0.145 (0.002)	0.613 (0.005)	0.144 (0.007)	0.595 (0.004)	0.144 (0.011)	0.732 (0.006)	0.147 (0.003)	0.749 (0.002)
	$L_{\infty }$	0.149 (0.005)	0.753 (0.002)	0.151 (0.003)	0.470 (0.002)	0.149 (0.002)	0.543 (0.003)	0.146 (0.004)	0.540 (0.003)	0.147 (0.011)	0.565 (0.003)	0.149 (0.002)	0.692 (0.005)	0.153 (0.007)	0.725 (0.005)
ResNet-15	-	0.168 (0.006)	0.621 (0.004)	0.172 (0.008)	0.418 (0.003)	0.169 (0.007)	0.461 (0.005)	0.165 (0.006)	0.488 (0.004)	0.167 (0.008)	0.508 (0.005)	0.171 (0.006)	0.601 (0.004)	0.173 (0.007)	0.628 (0.005)
PGDLM	$L_{\frac{1}{2}}$	0.177 (0.005)	0.577 (0.005)	0.185 (0.014)	0.502 (0.002)	0.179 (0.010)	0.473 (0.004)	0.171 (0.012)	0.520 (0.004)	0.188 (0.008)	0.548 (0.006)	0.175 (0.007)	0.463 (0.006)	0.182 (0.009)	0.691 (0.004)
LightGBM	$L_{1}$	0.194 (0.006)	0.561 (0.002)	0.203 (0.007)	0.491 (0.002)	0.198 (0.011)	0.464 (0.002)	0.189 (0.005)	0.493 (0.002)	0.211 (0.009)	0.533 (0.005)	0.199 (0.012)	0.461 (0.001)	0.205 (0.010)	0.661 (0.003)

In comparison, the convex regularizers $L_{1}$ and $L_{2}$ performed moderately well, with MSE values close to, but generally higher than, those of $L_{\frac{1}{2}}$. For example, for Trait 1, the $L_{2}$ regularizer yielded an MSE of $0.145 \pm 0.011$ compared with $0.141 \pm 0.007$ for $L_{\frac{1}{2}}$. This small but consistent performance gap suggests that fractional penalties offer a slight advantage by encouraging sparsity without being overly restrictive. Other regularizers, including the non-convex MCP and SCAD, and the higher-order $L_{p}$ norms ($L_{3}$, $L_{4}$, and $L_{\infty }$), performed notably worse. SCAD yielded some of the highest MSE values, such as $0.160 \pm 0.002$ for Trait 7, with MCP showing a similar degradation in predictive accuracy. The consistent underperformance of these regularizers across the mice, wheat, and pine datasets indicates that they may not be well suited for deeper ResNet architectures in genomic prediction. The higher-order $L_{p}$ norms also underperformed, yielding test MSEs in the range of $0.146\mathrm{--}0.155$. For instance, $L_{3}$ resulted in an MSE of $0.155 \pm 0.003$ for Trait 7, and $L_{\infty }$ gave $0.153 \pm 0.007$ for the same trait. This highlights the importance of using penalties that effectively encourage sparsity without losing critical information, a balance that the $L_{\frac{1}{2}}$ regularizer appears to strike exceptionally well. For the non-regularized ResNet-15, it achieved MSE values ranging from $0.165 \pm 0.006$ (Trait 4) to $0.173 \pm 0.007$ (Trait 7), substantially worse than all regularized variants. This demonstrated the advantage of the regularization method in this research. And both PGDLM with regularizer $L_{\frac{1}{2}}$ and the classical LightGMB generated higher test MSEs and lower dCor. All experimental results for the various ResNet architectures (ResNet-5, 10, 18, 20, and 25) evaluated on the pine dataset are provided in Supplementary Material S4.

### Comparison of sparsity and computing efficiency

**Analysis of sparsity:** The integration of non-convex regularizers within the ResNet architecture yields sparse network structure across the four datasets. As summarized in Table 5, the $L_\frac{1}{2}$ penalty consistently achieves the highest weight sparsity compared with both non-convex alternatives such as MCP and SCAD, and the convex $L_{1}$ regularizer, with sparsity levels ranging from 88.94% for pig to 90.93% for wheat. Despite this significant reduction in model complexity, these models simultaneously achieve superior predictive accuracy, suggesting that the $L_\frac{1}{2}$ penalty successfully identifies the most critical network weights by pruning redundant connections more effectively than $L_{1}$ or $L_{0}$ approaches.

**Table 5:** Weight sparsity (%) for ResNet-18 (mice and pig) and ResNet-15 (wheat and loblolly pine) with different regularizers across four genomic datasets. The values represent the percentage of network weights set to zero by the proximal operator; higher values indicate greater sparsity

**Regularizer**	**Mice**	**Pig**	**Wheat**	**Loblolly Pine**
$L_{0}$	84.91	84.24	85.27	85.12
$L_{\frac{1}{2}}$	89.83	88.94	90.93	89.35
$L_{\frac{2}{3}}$	85.87	85.45	86.19	86.13
MCP	85.29	84.99	86.67	86.19
SCAD	84.97	84.24	85.89	85.47
$L_{1}$	87.81	86.38	87.91	86.85

Moreover, the comparative analysis reveals a distinct divergence in feature selection between the $L_\frac{1}{2}$-regularized ResNet and the traditional $L_{1}$ penalty, which achieves moderate sparsity with more redundant features. Hence, the $L_\frac{1}{2}$ penalty demonstrates a superior ability to identify informative markers. To further characterize the selected features, we examined the MAF distribution of selected versus non-selected SNPs across all four datasets. The Mann–Whitney $U$ test results show that there is no significant difference in MAF between selected and nonselected SNPs, suggesting that the $L_\frac{1}{2}$-regularized ResNet identifies informative features through learned representations rather than by exploiting allele frequency (Mice $P=0.567$, Pig $P=0.446$, Wheat $P=0.463$, Loblolly Pine $P=0.458$). To evaluate the mechanistic contribution of the proposed adaptive proximal gradient method and the informativeness of the selected features, we conducted a dual-factor ablation study across all four datasets. Our ablation study highlights three key findings: (i) the $L_{\frac{1}{2}}$ proximal operator is indispensable for framework optimization; (ii) feature selection is highly effective, as subsets representing only $5.3\%$ of markers outperform the complete dataset; and (iii) proximal regularization enables the identification of informative SNPs regardless of underlying population structure. The method and experimental results and related analysis have been provided in the Supplementary Material S5. Trait-specific weight sparsity comparisons across all four datasets are provided in Supplementary Materials S6 and S7.

**Computing efficiency analysis:** The results presented in Table 5 highlight the distinct impacts of various regularizers on model architecture and their generalization properties. Across the four genomic datasets, the $L_\frac{1}{2}$ regularizer consistently achieved an optimal balance between model architecture and predictive power. We can see that $L_\frac{1}{2}$ regularization induced the most compact architectural state, with sparsity levels localized between 9% and 11%, representing a significant reduction in model complexity compared with the 14%–15% density observed in the other non-convex alternatives. This suggests that the $L_\frac{1}{2}$ norm exerts a more aggressive and precise shrinkage on the parameter space, effectively pruning non-informative markers while preserving the essential marker signals. While convex baselines such as $L_{1}$ facilitated moderate parameter reduction, they were outperformed in both predictive stability and feature selection resolution. Although MCP and SCAD provided some level of shrinkage, their lower sparsity efficiency often correlated with unstable generalization. Furthermore, the assessment of computational efficiency shows that training time ranged from $\sim $30 min for the Wheat data to nearly 200 min for the Pig data. It means that the choice of regularizer has little effect on total execution time for a fixed model depth. Hence, the fractional penalties, specifically the $L_\frac{1}{2}$ norm, provide a flexible and computationally scalable framework for genomic prediction, yielding highly sparse models that retain maximum signal without additional computational cost.

### Analysis of statistical significance and architectural depth

To assess whether the performance advantage of the $L_\frac{1}{2}$-regularized ResNet over the non-regularized baseline is statistically significant across all architectures, we applied the Wilcoxon signed-rank test to the 10 paired test MSE values per trait for each ResNet depth (ResNet-5 through ResNet-25) on all four datasets. Results show that (i) for shallow architectures such as ResNet-5, $P$-values stay near the significance threshold ($\alpha = 0.05$), suggesting that the structural bottleneck of shallow networks imposes a form of implicit regularization that reduces the marginal benefit of the explicit proximal penalty, and (ii) as architectural depth increases to ResNet-20 and ResNet-25, $P$-values decay considerably across all the datasets, indicating that the performance advantage of $L_\frac{1}{2}$ regularization becomes increasingly important with network depth. This trend is consistent across datasets with substantially different characteristics (sample size, marker density, trait complexity), showing that the finding reflects a general property of the proximal regularization framework rather than a specified dataset.

We can also see that as network depth increases, the ratio of trainable parameters to observations grows substantially, creating conditions conducive to noise memorization in the absence of explicit regularization [49, 50]. We can conclude that the best $L_\frac{1}{2}$ regularizer can mitigate this problem by concentrating predictive weights on a sparse subset of informative markers, suppressing model complexity and improve the performance of ResNets. Compared with the convex $L_{1}$ norm, the non-convex $L_\frac{1}{2}$ penalty exerts a more aggressive sparsity-inducing behavior, as confirmed by the higher weight sparsity values reported in Table 5. These results confirm the important role of the non-convex sparse penalties in stabilizing high-dimensional deep learning models. Detailed Wilcoxon test results for each dataset are provided in Supplementary Material S7.

## Conclusion

In this study, we introduce a flexible proximal regularization framework for deep residual neural networks and demonstrate its effectiveness for genomic prediction across four high-dimensional datasets. By integrating convex and non-convex proximal operators into an adaptive gradient scheme, the proposed approach can control model complexity, promote sparsity, and improve generalization in settings where marker dimensionality greatly exceeds sample size. Our empirical analyses show that moderately deep architectures, such as ResNet-18 and ResNet-15, consistently improve predictive accuracy, while the $L_{\frac{1}{2}}$ regularizer yields the lowest test MSEs and highest distance correlations. Compared with traditional sparse linear models with the same regularizers, non-regularized ResNets, and a competitive machine learning baseline (LightGBM), the regularized ResNet framework achieves more accurate and stable predictions across four real datasets, highlighting the value of combining deep architectures with structured proximal regularization for modeling complex genotype–phenotype relationships in breeding and precision medicine.

**Key Points**

When trained on small, noisy, and high-dimensional data, ResNets will suffer from overfitting due to the large amount of parameters. One promising regularization approach relies on the proximal mapping technique, which is computationally efficient since it can be directly incorporated into the optimization algorithm. However, the performance of ResNets with various convex or non-convex proximal regularizers remains under-explored on high-dimensional data. In this study, we present a stochastic adaptive proximal gradient ResNet method with penalty functions spanning from convex to non-convex regularizers (between $L_{0}$ and $L_{\infty }$). The key points in this research include:

- Methodological advancement: the development of the extended stochastic adaptive proximal gradient ResNet capable of handling diverse regularizers, providing a flexible framework for sparse and efficient deep learning.
- Theoretical analysis and guarantee: a detailed analysis and derivation of the convergence guarantee for both the convex and non-convex regularizers within the proposed framework.
- Empirical validation: comprehensive evaluation of the method’s prediction performance in a supervised regression setting using four real, high-dimensional genomic datasets (mice, pig, wheat, and loblolly pine).

## Supplementary Material

supplementary_files_bbag246

## Data Availability

The original data sets are available at: Mice data: https://cran.r-project.org/web/packages/BGLR/ Pig data: https://academic.oup.com/g3journal/article/2/4/429/6026060/ Wheat data: https://cran.r-project.org/web/packages/BGLR/index.html Loblolly pine data: https://academic.oup.com/genetics/article/190/4/1503/6064084/. All the data and the code are available online at https://github.com/angelYHF/Adaptive-gradient-methods.
