# Supplementary material for: Proximal regularization of deep residual neural networks applied to high-dimensional genomic data
Source: Brief Bioinform. 2026 May 25;27(3):bbag246. doi: 10.1093/bib/bbag246 (PMC13200547; doi:10.1093/bib/bbag246)
Supplement: supplementary_files_bbag246 [file supplementary_files_bbag246.zip › Appendices_for_the_submission_BIB_25_2359_updated.pdf]

Journal Title Here, 2022, pp. 1–4

doi: DOI HERE
Advance Access Publication Date: Day Month Year
Paper

Supplementary material for the submission BIB-25-2359

PAPER

Proximal Regularization of Deep Residual Neural Networks Applied to High-dimensional Genomic Data

Yuhua Fan,[1] Ilkka Launonen,[1] Mikko J. Sillanpää[1] and Patrik Waldmann[1,*]

[1]Research Unit of Mathematical Sciences, University of Oulu, Oulu, Finland
[*]Corresponding author. Patrik.Waldmann@oulu.fi

Abstract

Motivation: Residual neural networks (ResNets) offer strong representational capacity for modeling complex genomic relationships, but their large parameter space makes them susceptible to overfitting in high-dimensional settings. Proximal regularization provides an attractive solution because it allows non-smooth or non-convex penalties to be incorporated directly into gradient-based optimization. Despite its promise, the behavior of ResNets under different convex and non-convex proximal operators has not been systematically analyzed for genomic prediction. When trained on small, noisy and high-dimensional data, ResNets may suffer from overfitting due to the large amount of parameters. As a solution, a range of regularization methods have been proposed. One promising approach relies on the proximal mapping technique which is computationally efficient since it can be directly incorporated into the optimization algorithm. However, the performance of ResNets with various convex or non-convex proximal regularizers remains under-explored on high-dimensional data. In this study, we propose an extended stochastic adaptive proximal gradient ResNet method that can handle both convex and non-convex regularizers that range from L_0 to L_∞ and give more analysis of the convergence guarantee for the convex and non-convex regularizers.
Supplementary overview: This supplementary document provides the detailed methodological foundations underlying the adaptive proximal gradient ResNet framework presented in the main manuscript. We give full derivations of the proximal operators for a wide family of convex and non-convex penalties ranging from L_0 to L_∞, including fractional, folded-concave, and hard-thresholding-based regularizers. In addition, we present theoretical results characterizing the convergence properties of the proposed stochastic adaptive proximal gradient algorithm under both convex and non-convex settings. These analyses complement the empirical findings in the main text and offer deeper insight into why structured proximal regularization can improve stability and generalization in high-dimensional genomic prediction. Notice that we focus specifically on rational values of q (expressed as the ratio of two integers) rather than general real-valued exponents.
Results: In this study, we propose an extended stochastic adaptive proximal gradient ResNet method that can handle both convex and non-convex regularizers that range from L_0 to L_∞ and give more analysis of the convergence guarantee for the convex and non-convex regularizers. Moreover, we evaluate the prediction performance in a supervised regression setting on four real high-dimensional genomic datasets from mice, pig, wheat and loblolly pine. For comparison, we also implement and evaluate traditional sparse linear proximal methods with the same regularizers, as well as LightGBM. Experimental results demonstrate that an 18-layer ResNet with $L_{\frac{1}{2}}$ regularization outperforms other configurations on both mice and pig datasets. For the wheat and loblolly pine data, the 15-layer ResNet $L_{\frac{1}{2}}$ configuration achieves the lowest test mean squared errors. These findings highlight the effectiveness of the regularized adaptive proximal gradient ResNet method and its potential for prediction tasks on high-dimensional genomic data.
Availability: All the data and the code are available online at: https://github.com/angelYHF/Adaptive-gradient-methods.
Contact: patrik.waldmann@oulu.fi
Supplementary information: This document is for the submission BIB-25-2359. Two sections are included: (1) proximal operator from L_0 to L_∞ and their i-th coordinate update with preconditioner. (2) A comprehensive analysis of convergence guarantee for the algorithm.

Proximal Mappings of The Regularization Functions

Here we present the proximal operators for the regularizers L_0 to L_∞ and how they are combined with the Adam preconditioners presented in Section 2 (Methods). We also consider the cases of MCP and SCAD regularization. Recall that the minimization problem for L_q regularizers is

$$\arg\min_{\theta} \left\{ \frac{1}{2} \left\| \theta - \hat{\theta}_t \right\|^2_{C_t+\delta I} + \lambda \sum_{j=1}^{p} |\theta_j|^q \right\}, \tag{1}$$

for which a solution is obtained by iterating

$$\begin{aligned} \hat{\theta}_t &= \theta_t - \alpha_t (C_t + \delta I)^{-1} m_t, \\ \theta_{t+1} &= \text{prox}^{C_t+\delta I}_{\alpha_t \lambda \mathcal{R}(\cdot)}(\hat{\theta}_t). \end{aligned} \tag{2}$$

By denoting the i-th coordinate of θ_t as $\theta_{t,i}$ and the i-th diagonal entry of the preconditioner $[C_t]_{ii}$ as $C_{t,i}$, the formulation (2) is coordinate-wise decomposable and the preconditioner matrix C_t is diagonal, so we get

$$\begin{aligned} \theta_{t+1,i} &= \arg\min_{\theta_i} \left\{ \frac{1}{2}(C_{t,i} + \delta I)(\theta_i - \hat{\theta}_{t,i})^2 + \alpha_t \lambda |\theta_i|^q \right\} \\ &= \arg\min_{\theta_i} \left\{ (\theta_i - \hat{\theta}_{t,i})^2 + \frac{2\alpha_t \lambda}{C_{t,i} + \delta I} |\theta_i|^q \right\}, \end{aligned} \tag{3}$$

where

$$\hat{\theta}_{t,i} = \theta_{t,i} - \alpha_t \frac{m_{t,i}}{C_{t,i} + \delta I}. \tag{4}$$

L_0 Regularization

When $q = 0$ in the regularization term, the penalty function is L_0, which penalizes the number of non-zero entries. The closed-form proximal operator is obtained via hard-thresholding:

$$\text{prox}_{\lambda \mathcal{R}(\cdot)}(\theta) = \begin{cases} \{0\}, & |\theta| < \sqrt{2\lambda}, \\ \{0, \theta\}, & |\theta| = \sqrt{2\lambda}, \\ \{\theta\}, & |\theta| > \sqrt{2\lambda}. \end{cases} \tag{5}$$

The closed-form proximal mappings for L_0 regularization combined with the preconditioner $C_{t,i}$ lead to the following update rule (1):

$$\theta_{t+1,i} = \begin{cases} \hat{\theta}_{t,i}, & \text{if } \left|\hat{\theta}_{t,i}\right| > \sqrt{\frac{2\alpha_t\lambda}{C_{t,i}+\delta I}}, \\ 0, & \text{if } \left|\hat{\theta}_{t,i}\right| < \sqrt{\frac{2\alpha_t\lambda}{C_{t,i}+\delta I}}, \\ \left\{0, \hat{\theta}_{t,i}\right\}, & \text{if } \left|\hat{\theta}_{t,i}\right| = \sqrt{\frac{2\alpha_t\lambda}{C_{t,i}+\delta I}}. \end{cases} \tag{6}$$

$L_{\frac{1}{2}}$ Regularization

For $q = \frac{1}{2}$, the $L_{\frac{1}{2}}$ quasinorm proximal mapping (2) is:

$$\text{prox}_{\lambda \mathcal{R}(\cdot)}(\theta) = \begin{cases} \frac{2}{3}|\theta|\left(1 + \cos\left(\frac{2}{3}\pi - \frac{2}{3}\varphi_\lambda(\theta)\right)\right), & \text{if } \theta > p(\lambda), \\ 0, & \text{if } |\theta| \le p(\lambda) \\ -\frac{2}{3}|\theta|\left(1 + \cos\left(\frac{2}{3}\pi - \frac{2}{3}\varphi_\lambda(\theta)\right)\right), & \text{if } \theta < -p(\lambda) \end{cases} \tag{7}$$

where $\varphi_\lambda(\theta) = \arccos\left(\frac{\lambda}{4}\left(\frac{\theta}{3}\right)^{-\frac{3}{2}}\right)$ and $p(\lambda) = \frac{\sqrt[3]{54}}{4}(\lambda)^{\frac{2}{3}}$. The i-th coordinate update with preconditioner is:

$$\theta_{t+1,i} = \begin{cases} \frac{2}{3}\left|\hat{\theta}_{t,i}\right|(1 + \cos(\frac{2}{3}\pi - \frac{2}{3}\varphi_\lambda(\hat{\theta}_{t,i}))), & \text{if } \hat{\theta}_{t,i} > p(\lambda) \\ 0, & \text{if } \left|\hat{\theta}_{t,i}\right| \le p(\lambda) \\ -\frac{2}{3}\left|\hat{\theta}_{t,i}\right|(1 + \cos(\frac{2}{3}\pi - \frac{2}{3}\varphi_\lambda(\hat{\theta}_{t,i}))), & \text{if } \hat{\theta}_{t,i} < -p(\lambda) \end{cases} \tag{8}$$

where

$$\varphi_\lambda(\hat{\theta}_{t,i}) = \arccos\left(\frac{\alpha_t\lambda}{4(C_{t,i}+\delta I)}\left(\frac{\left|\hat{\theta}_{t,i}\right|}{3}\right)^{-\frac{3}{2}}\right), \quad p(\lambda) = \frac{\sqrt[3]{54}}{4}\left(\frac{2\alpha_t\lambda}{C_{t,i}+\delta I}\right)^{\frac{2}{3}}. \tag{9}$$

$L_{\frac{2}{3}}$ Regularization

For $q = \frac{2}{3}$, the proximal operator (2) is:

$$\text{prox}_{\lambda\mathcal{R}(\cdot)}(\theta) = \begin{cases} \left(\frac{|A| + \sqrt{\frac{2|\theta|}{|A|} - |A|^2}}{2}\right)^3, & \text{if } \theta > \frac{2}{3}\sqrt[4]{3\lambda^3} \\ 0, & \text{if } |\theta| \le \frac{2}{3}\sqrt[4]{3\lambda^3} \\ -\left(\frac{|A| + \sqrt{\frac{2|\theta|}{|A|} - |A|^2}}{2}\right)^3, & \text{if } \theta < -\frac{2}{3}\sqrt[4]{3\lambda^3} \end{cases} \tag{10}$$

where

$$|A| = \frac{2}{\sqrt{3}}\lambda^{\frac{1}{4}}\left(\cosh\left(\frac{\phi}{3}\right)\right)^{\frac{1}{2}}, \quad \phi = \text{arccosh}\left(\frac{27\theta^2}{16}\lambda^{-\frac{3}{2}}\right). \tag{11}$$

The i-th coordinate update is:

$$\theta_{t+1,i} = \begin{cases} \left(\frac{|A| + \sqrt{\frac{2\left|\hat{\theta}_{t,i}\right|}{|A|} - |A|^2}}{2}\right)^3, & \text{if } \hat{\theta}_{t,i} > \frac{2}{3}\sqrt[4]{3\lambda^3} \\ 0, & \text{if } \left|\hat{\theta}_{t,i}\right| \le \frac{2}{3}\sqrt[4]{3\lambda^3} \\ -\left(\frac{|A| + \sqrt{\frac{2\left|\hat{\theta}_{t,i}\right|}{|A|} - |A|^2}}{2}\right)^3, & \text{if } \hat{\theta}_{t,i} < -\frac{2}{3}\sqrt[4]{3\lambda^3} \end{cases} \tag{12}$$

where

$$|A| = \frac{2}{\sqrt{3}}\left(\frac{2\alpha_t\lambda}{C_{t,i}+\delta I}\right)^{\frac{1}{4}}\left(\cosh\left(\frac{\phi}{3}\right)\right)^{\frac{1}{2}}, \quad \phi = \text{arccosh}\left(\frac{27\hat{\theta}^2_{t,i}}{16}\left(\frac{2\alpha_t\lambda}{C_{t,i}+\delta I}\right)^{-\frac{3}{2}}\right). \tag{13}$$

MCP Regularization

The proximal mapping of the MCP (Minimax Concave Penalty) (3) is:

$$\text{prox}_{\lambda\mathcal{R}(\cdot)}(\theta) = \begin{cases} 0, & |\theta| \le \lambda \\ \frac{\text{sign}(\theta)(|\theta| - \lambda)}{1 - \frac{1}{a}}, & \lambda < |\theta| \le a\lambda \\ \theta, & |\theta| > a\lambda \end{cases} \tag{14}$$

The i-th coordinate update (with $a = 2$) is:

$$\theta_{t+1,i} = \text{sign}(\hat{\theta}_{t,i})\min\left\{\frac{a\max\left\{\left|\hat{\theta}_{t,i}\right| - \frac{\alpha_t\lambda}{C_{t,i}+\delta I}, 0\right\}}{a-1}, \left|\hat{\theta}_{t,i}\right|\right\}. \tag{15}$$

SCAD Regularization

The proximal mapping of the SCAD (Smoothly Clipped Absolute Deviation) penalty (3) is:

$$\text{prox}_{\lambda\mathcal{R}(\cdot)}(\theta) = \begin{cases} \text{sign}(\theta)\max\{|\theta - \lambda|, 0\}, & \text{if } |\theta| \le 2\lambda \\ \frac{(a-1)\theta - \text{sign}(\theta)a\lambda}{a-2}, & \text{if } 2\lambda < |\theta| \le a\lambda \\ \theta, & \text{if } |\theta| > a\lambda, \end{cases} \tag{16}$$

The i-th coordinate update (with $a = 3.7$) is:

$$\theta_{t+1,i} = \begin{cases} \text{sign}(\hat{\theta}_{t,i})\max\left\{\left|\hat{\theta}_{t,i}\right| - \hat{\lambda}_i, 0\right\}, & \text{if } \left|\hat{\theta}_{t,i}\right| \le 2\hat{\lambda}_i \\ \frac{(a-1)\hat{\theta}_{t,i} - \text{sign}(\hat{\theta}_{t,i})a\hat{\lambda}_i}{a-2}, & \text{if } 2\hat{\lambda}_i < \left|\hat{\theta}_{t,i}\right| \le a\hat{\lambda}_i \\ \hat{\theta}_{t,i}, & \text{if } \left|\hat{\theta}_{t,i}\right| > a\hat{\lambda}_i \end{cases} \tag{17}$$

where $\hat{\lambda}_i = \frac{\alpha_t\lambda}{C_{t,i}+\delta I}$.

© The Author 2022. Published by Oxford University Press. All rights reserved. For permissions, please e-mail: journals.permissions@oup.com

L_1 Regularization

For $q = 1$, the L_1 norm proximal mapping (4) is the soft-thresholding operator:

$$\begin{aligned} \text{prox}_{\lambda\mathcal{R}(\cdot)}(\theta) &= \text{sign}(\theta)\max(|\theta| - \lambda, 0) \\ &= \text{sign}(\theta)(|\theta| - \lambda)_+, \end{aligned} \tag{18}$$

The i-th coordinate update with preconditioner is:

$$\theta_{t+1,i} = \text{sign}(\hat{\theta}_{t,i}) \left(\left|\hat{\theta}_{t,i}\right| - \frac{\alpha_t \lambda}{C_{t,i} + \delta} \right). \tag{19}$$

$L_{\frac{4}{3}}$ Regularization

For $q = \frac{4}{3}$, the proximal mapping (5) is:

$$\text{prox}_{\lambda\mathcal{R}(\cdot)} = \theta + \frac{4\lambda\kappa}{32^{\frac{1}{3}}} \left((\chi - \theta)^{\frac{1}{3}} - (\chi + \theta)^{\frac{1}{3}} \right), \tag{20}$$

where $\chi = \sqrt{\theta^2 + 256\kappa^3/729}$ and $\kappa = 1$. The i-th coordinate update rule is:

$$\theta_{t+1,i} = \hat{\theta}_{t,i} + \frac{8\kappa\alpha_t\lambda}{32^{\frac{1}{3}}(C_{t,i} + \delta I)}((\chi - \hat{\theta}_{t,i})^{\frac{1}{3}} - (\chi + \hat{\theta}_{t,i})^{\frac{1}{3}}), \tag{21}$$

where $\chi = \sqrt{\hat{\theta}_{t,i}^2 + 256\kappa^3/729}$.

$L_{\frac{3}{2}}$ Regularization

For $q = \frac{3}{2}$, the proximal mapping (5) is:

$$\text{prox}_{\lambda\mathcal{R}(\cdot)} = \theta + 9\kappa^2 \text{sgn}\left(\theta\right) \left(1 - \sqrt{1 + 16\lambda \left|\theta\right| / (9\kappa^2)} \right) /8, \tag{22}$$

where $\kappa = 1$. The i-th coordinate update rule is:

$$\theta_{t+1,i} = \hat{\theta}_{t,i} + 9\kappa^2 \text{sgn}(\hat{\theta}_{t,i}) \left(1 - \sqrt{1 + \frac{32\left|\hat{\theta}_{t,i}\right|\alpha_t\lambda}{9\kappa^2(C_{t,i} + \delta I)}} \right) /8. \tag{23}$$

L_2 Regularization

When $q = 2$, the L_2 norm proximal mapping (4) is:

$$\text{prox}_{\lambda\mathcal{R}(\cdot)} = \left(1 - \frac{\lambda}{\|\theta\|_2} \right)_+ \theta, \tag{24}$$

where $(\theta)_+ = \max\{0, \theta\}$. The i-th coordinate update with preconditioner is:

$$\theta_{t+1,i} = \left(1 - \frac{2\alpha_t\lambda}{(C_{t,i} + \delta I)\left\|\hat{\theta}_{t,i}\right\|_2} \right)_+ \hat{\theta}_{t,i}. \tag{25}$$

L_3 Regularization

For $q = 3$, the L_3 norm proximal mapping (5) is:

$$\text{prox}_{\lambda\mathcal{R}(\cdot)} = \text{sgn}(\theta)(\sqrt{1 + 12\lambda\kappa\left|\theta\right|} - 1)/(6\kappa), \tag{26}$$

where $\kappa = 1$. The i-th coordinate update with preconditioner is:

$$\theta_{t+1,i} = \text{sgn}(\hat{\theta}_{t,i}) \frac{\sqrt{1 + \frac{24\kappa\alpha_t\lambda\left|\hat{\theta}_{t,i}\right|}{C_{t,i} + \delta I}} - 1}{6\kappa}. \tag{27}$$

L_4 Regularization

For $q = 4$, the L_4 norm proximal mapping (5) is:

$$\text{prox}_{\lambda\mathcal{R}(\cdot)} = \left(\frac{\chi + \lambda\theta}{8\kappa} \right)^{\frac{1}{3}} - \left(\frac{\chi - \lambda\theta}{8\kappa} \right)^{\frac{1}{3}}, \tag{28}$$

where $\kappa = 1$ and $\chi = \sqrt{\theta^2 + \frac{1}{27\kappa}}$. The i-th coordinate update rule is:

$$\theta_{t+1,i} = \left(\frac{\chi + \frac{2\hat{\theta}_{t,i}\alpha_t\lambda}{C_{t,i} + \delta I}}{8\kappa} \right)^{\frac{1}{3}} - \left(\frac{\chi - \frac{2\hat{\theta}_{t,i}\alpha_t\lambda}{C_{t,i} + \delta I}}{8\kappa} \right)^{\frac{1}{3}}, \tag{29}$$

where $\chi = \sqrt{\hat{\theta}_{t,i}^2 + \frac{1}{27\kappa}}$.

L_∞ Regularization

For the infinity norm, the i-th coordinate update rule is (4):

$$\theta_{t+1,i} = \begin{cases} \hat{\theta}_{t,i} - \frac{2\alpha_t\lambda}{C_{t,i} + \delta I}, & \hat{\theta}_{t,i} > \frac{2\alpha_t\lambda}{C_{t,i} + \delta}, \\ \hat{\theta}_{t,i} + \frac{2\alpha_t\lambda}{C_{t,i} + \delta}, & \hat{\theta}_{t,i} < \frac{2\alpha_t\lambda}{C_{t,i} + \delta}, \\ 0, & \text{otherwise.} \end{cases} \tag{30}$$

Analysis of Convergence Guarantee

This section provides a concise analysis of the theoretical convergence guarantees for our adaptive proximal gradient methods, considering both convex and non-convex regularizers.

Convex Regularizers

For the convex case, we examine the conditions that guarantee convergence based on standard arguments from the convex optimization literature (6; 4). The objective function is $\mathcal{F}(\theta) = \mathcal{L}(\theta) + \lambda\mathcal{R}(\theta)$, where both $\mathcal{L}$ and $\mathcal{R}$ are convex.

Assumptions for Convergence

1. **Convexity:** The loss function $\mathcal{L}(\theta)$ is convex and continuously differentiable. The regularizer $\mathcal{R}(\theta)$ is also convex.
2. ***L*-Smoothness:** The loss function $\mathcal{L}(\theta)$ is L-smooth, meaning its gradient is Lipschitz continuous:

$$\|\nabla\mathcal{L}(\theta) - \nabla\mathcal{L}(\theta')\| \leq L\|\theta - \theta'\|. \tag{31}$$

3. **Existence of a minimum:** The set of minimizers, $\Theta^* = \arg\min_\theta \mathcal{F}(\theta)$, is non-empty.

Convergence Guarantee

For a standard proximal gradient descent with a constant learning rate $\alpha < 1/L$, the convergence rate is typically sublinear:

$$\mathcal{F}(\theta_T) - \mathcal{F}(\theta^*) \leq \frac{1}{T} \left(\frac{\|\theta_0 - \theta^*\|^2}{2\alpha} \right). \tag{32}$$

Our adaptive method often achieves faster empirical convergence than standard proximal gradient methods, though theoretical guarantees require additional conditions, as discussed in (7).

Non-Convex Regularizers

For non-convex optimization, the goal is to find a stationary point.

Assumptions for Convergence

1. **Differentiability and Lipschitz continuity:** The loss function $\mathcal{L}(\theta)$ is continuously differentiable and L-smooth.
2. **Lower bounded:** The objective function $\mathcal{F}(\theta)$ is bounded from below.
3. **Sufficient decrease:** The algorithm ensures a sufficient decrease in the objective function at each step.

Convergence Guarantee to an ϵ-Stationary Point

The convergence is measured by the norm of the limiting subdifferential. A common result is that the average squared norm of the subgradient is bounded:

$$\min_{t=1,\dots,T} \|\partial \mathcal{F}(\theta_t)\|^2 \leq \frac{\mathcal{F}(\theta_1) - \mathcal{F}(\theta_T)}{\sum_{t=1}^{T} \alpha_t} \quad (33)$$

where $\partial \mathcal{F}(\theta_t)$ denotes a subgradient. This implies convergence to a stationary point. The adaptive proximal gradient approach, similar to ProxGEN (1), is guaranteed to converge to a stationary point under specific parameter choices and boundedness assumptions on the step-vector, stochastic gradient, and effective spectrum eigenvalue (8; 9). The key parameter requirements are: (i) final step-vector finite, (ii) stochastic gradient bounded, and (iii) minimum effective spectrum eigenvalue uniformly lower bounded.

Competing interests

No competing interest is declared.

Author contributions statement

Yuhua Fan and Partrik Waldmann conceived the experiment(s), Yuhua Fan conducted the experiment(s), Yuhua Fan, Ilkka Launonen, Mikko Sillanpää and Patrik Waldmann analysed the results and wrote the manuscript.

Acknowledgments

This work was supported by the Academy of Finland Prof 5 funding for mathematics and AI: data insight for high dimensional dynamics [Grant 326291].

Data Availability Statement

The original data sets are available at:
Mice data:
https://cran.r-project.org/web/packages/BGLR/
Pig data:
https://academic.oup.com/g3journal/article/2/4/429/6026060/
Wheat data:
https://cran.r-project.org/web/packages/BGLR/index.html
Loblolly pine data:
https://academic.oup.com/genetics/article/190/4/1503/6064084/

Key Points

When trained on small, noisy and high-dimensional data, ResNets will suffer from overfitting due to the large amount of parameters. For the the existing methods, one promising approach relies on the proximal mapping technique which is computationally efficient since it can be directly incorporated into the optimization algorithm. However, the performance of ResNets with various convex or non-convex proximal regularizers remains under-explored on high-dimensional data. In this study, an extended stochastic adaptive proximal gradient ResNet method is pr. This innovative approach is computationally efficient and can successfully incorporate a wide range of regularization techniques, spanning from convex to non-convex regularizers (from L_0 to L_∞). The key points in this research include:

- Methodological advancement: The development of the extended stochastic adaptive proximal gradient ResNet capable of handling diverse regularizers, providing a flexible framework for sparse and efficient deep learning.
- Theoretical analysis and guarantee: A detailed analysis and derivation of the convergence guarantee for both the convex and non-convex regularizers within this proposed framework.
- Empirical validation: Comprehensive evaluation of the method's prediction performance in a supervised regression setting using four real, high-dimensional genomic datasets (mice, pig, wheat, and loblolly pine).

References

1. Jihun Yun, Aurélie C Lozano, and Eunho Yang. Adaptive proximal gradient methods for structured neural networks. *Advances in Neural Information Processing Systems*, 34:24365–24378, 2021.
2. Wenfei Cao, Jian Sun, and Zongben Xu. Fast image deconvolution using closed-form thresholding formulas of lq (q= 12, 23) regularization. *Journal of Visual Communication and Image Representation*, 24(1):31–41, 2013.
3. Yaohua Hu, Xinlin Hu, and Xiaoqi Yang. On convergence of iterative thresholding algorithms to approximate sparse solution for composite nonconvex optimization. *Mathematical Programming*, pages 1–26, 2024.
4. Neal Parikh and Stephen Boyd. Proximal algorithms. *Foundations and Trends in Optimization*, 1(3):127–239, 2014.
5. Nicholas G Polson, James G Scott, and Brandon T Willard. Proximal algorithms in statistics and machine learning. *Statistical Science*, 30(4):559–581, 2015.
6. Stephen Boyd and Lieven Vandenberghe. *Convex optimization*. Cambridge university press, 2004.
7. Sashank J Reddi, Satyen Kale, and Sanjiv Kumar. On the Convergence of Adam and Beyond. In *International Conference on Learning Representations*, 2018.
8. Congliang Chen, Li Shen, Fangyu Zou, and Wei Liu. Towards practical Adam: Non-convexity, convergence theory, and mini-batch acceleration. *Journal of Machine Learning Research*, 23:1–47, 2022.
9. Alexandre Défossez, Leon Bottou, Francis Bach, and Nicolas Usunier. A simple convergence proof of Adam and Adagrad. *Transactions on Machine Learning Research*, 2022.
