# Supplementary material for: Proximal regularization of deep residual neural networks applied to high-dimensional genomic data
Source: Brief Bioinform. 2026 May 25;27(3):bbag246. doi: 10.1093/bib/bbag246 (PMC13200547; doi:10.1093/bib/bbag246)
Supplement: supplementary_files_bbag246 [file supplementary_files_bbag246.zip › S5_bbag246.pdf]

Supplementary Material for Ablation Study and Informativeness Analysis

1 Method

To evaluate the mechanistic contribution of the proposed adaptive proximal gradient method and the informativeness of the selected features, we conducted a dual-factor ablation study across all four datasets: (1) the level of proximal operator coverage, evaluated at 0% (standard Adam, no proximal update), 50% (hybrid proximal), and 100% (full proximal updates); (2) the feature set used for training, comparing the complete genomic marker set against the informative subset identified via the $L_{1/2}$ regularizer.

2 Results and Analysis

Series of experimental results showed that:

- The integration of the non-convex $L_{\frac{1}{2}}$ proximal operator with ResNet is fundamental to model optimization. We observed an enhancement in predictive accuracy as the proportion of proximal updates increased. From the Table 1, we can see that for Mice Trait 1, the dCor rose from 0.521 with 0% proximal updates to 0.638 at 50%, ultimately reaching 0.733 with full proximal integration. This confirms that the adaptive gradient method effectively navigates the complex, non-convex loss landscape to identify more optimal weight configurations than standard first-order optimizers.

- The informative subsets consistently outperformed the full genomic suites across all optimization settings and species. Notice that under full proximal updates for the Mice data, a model trained on only 5.3% of the total markers achieved a dCor of 0.761, significantly exceeding the 0.733 achieved by the full genomic set. Consistent improvements were observed across all four datasets after removing the majority of genomic background markers identified as non-informative by the $L_{1/2}$ regularizer: 94.7% for mice, 86.5% for pig, 90.5% for wheat, and 82.9% for loblolly pine (Table 1), confirming that the retained markers constitute a compact and informative feature representation for genomic prediction.

- to assess whether the selected markers are confounded by population structure, we examined their overlap with structure-associated SNPs identified via principal component analysis (PCA) of the full SNP matrix. The top 1% of SNPs by loading on the first 5 principal components were classified as structure-associated [1]. Structure overlap is defined as the percentage of selected SNPs (those with non-zero weights under the $L_{1/2}$ regularizer) that co-localize with these structure-associated markers. By construction of the 1% threshold, the overlap expected under random SNP selection is 1.0%. The observed overlap was at or below random expectation across all four datasets: 0.9% for mice, 1.0% for pig, 0.8% for wheat, and 1.1% for pine (Table 1), indicating that the $L_{1/2}$ regularizer selects SNPs independently of population stratification. This is consistent with [2], who demonstrated that appropriately regularized deep learning models can identify biologically informative SNPs that are largely independent of population structure. This finding is further corroborated by the MAF analysis (Table 1), which showed no significant difference in allele frequency between selected and non-selected SNPs across all four datasets.

References

[1] Price, Alkes L., et al. Principal components analysis corrects for stratification in genome-wide association studies. *Nature Genetics*, 38(8): 904-909, 2006.

[2] Dagasso, Gabrielle, et al. Accounting for population structure in deep learning models for genomic analysis. *Journal of Biomedical Informatics*, 169: 104873, 2025.

Table 1: Characterization of SNP selection by $L_{1/2}$-regularized ResNet across four genomic datasets.

**Dataset**	**Total SNPs**	**Selected SNPs**		**Sparsity (%)**	**Structure Overlap**		**MAF Comparison**		
		**Count**	**(%)**		**Count**	**(% of selected)**	**Selected**	**Non-selected**	***p*-value**
Mice	10,346	552	5.3	94.7	5	0.9	0.280	0.284	0.567
Pig	52,843	7,157	13.5	86.5	69	1.0	0.243	0.241	0.446
Wheat	1,279	121	9.5	90.5	1	0.8	0.258	0.249	0.463
Loblolly Pine	4,853	828	17.1	82.9	9	1.1	0.157	0.154	0.458
